# Cloning and Expression of Synthetic Genes Encoding the Broad Antimicrobial Spectrum Bacteriocins SRCAM 602, OR-7, E-760, and L-1077, by Recombinant *Pichia pastoris*

**DOI:** 10.1155/2015/767183

**Published:** 2015-03-02

**Authors:** Sara Arbulu, Juan J. Jiménez, Loreto Gútiez, Luis M. Cintas, Carmen Herranz, Pablo E. Hernández

**Affiliations:** Departamento de Nutrición, Bromatología y Tecnología de los Alimentos, Facultad de Veterinaria, Universidad Complutense de Madrid (UCM), Avenida Puerta de Hierro, s/n, 28040 Madrid, Spain

## Abstract

We have evaluated the cloning and functional expression of previously described broad antimicrobial spectrum bacteriocins SRCAM 602, OR-7, E-760, and L-1077, by recombinant *Pichia pastoris*. Synthetic genes, matching the codon usage of *P. pastoris*, were designed from the known mature amino acid sequence of these bacteriocins and cloned into the protein expression vector pPICZ*α*A. The recombinant derived plasmids were linearized and transformed into competent *P. pastoris* X-33, and the presence of integrated plasmids into the transformed cells was confirmed by PCR and sequencing of the inserts. The antimicrobial activity, expected in supernatants of the recombinant *P. pastoris* producers, was purified using a multistep chromatographic procedure including ammonium sulfate precipitation, desalting by gel filtration, cation exchange-, hydrophobic interaction-, and reverse phase-chromatography (RP-FPLC). However, a measurable antimicrobial activity was only detected after the hydrophobic interaction and RP-FPLC steps of the purified supernatants. MALDI-TOF MS analysis of the antimicrobial fractions eluted from RP-FPLC revealed the existence of peptide fragments of lower and higher molecular mass than expected. MALDI-TOF/TOF MS analysis of selected peptides from eluted RP-FPLC samples with antimicrobial activity indicated the presence of peptide fragments not related to the amino acid sequence of the cloned bacteriocins.

## 1. Introduction 

The antimicrobial peptides (AMPs) are broad spectrum small molecular weight compounds with antagonistic activity against bacteria, viruses, and fungi. Among them, bacteriocins form a widely studied and well-characterized group of ribosomally synthesized peptides produced by bacteria, and those produced by lactic acid bacteria (LAB) attract considerable interest primarily as natural food preservatives but also with great interest in exploring their application as therapeutic antimicrobial agents [1–3]. Most LAB bacteriocins are synthesized as biologically inactive precursors or prepropeptides containing an N-terminal extension that is cleaved off during export to generate their biologically active or mature form. The mature peptides are often cationic, amphiphilic molecules that are generally classified into two main classes: the lantibiotics or class I bacteriocins that consist of modified bacteriocins and the class II or nonmodified bacteriocins [4, 5]. The class II bacteriocins have been further divided into several subgroups, from which the class IIa or pediocin-like bacteriocins show a strong antilisterial activity and the N-terminal consensus sequence YGNGV(X)C [5].

Considering the low amount of AMPs obtained from their direct purification from natural producers and the elevated production costs of chemical synthesis, the biological production of bacteriocins by heterologous microbial hosts may provide an opportunity for their production in large amounts and with higher specific antimicrobial activity [6–8]. Furthermore, the production of bacteriocins by yeasts may have some advantages over bacterial cells regarding specific posttranscriptional and posttranslational modifications [9]. Yeasts are also cost-effective producers with large production and high yields of the desired protein [10].* Pichia pastoris* (currently reclassified as* Komagataella pastoris*) is being used as heterologous producer of bacteriocins because of its ability to produce large amounts of properly folded and biologically active bacteriocins [8, 11, 12].* P. pastoris* also produces disulfide-bonded and glycosylated proteins, which are crucial features for functionality [13]. The use of synthetic genes may also constitute a successful approach for heterologous production and functional expression of bacteriocins by recombinant yeasts when the DNA sequence encoding the bacteriocin is not available or difficult to obtain [12]. Furthermore, the use of synthetic genes matching the codon usage of the host microorganism can have a significant impact on gene expression levels and protein folding [14].

Several bacteriocins with broad antimicrobial spectrum have been identified from chicken commensal bacteria including bacteriocin SRCAM 602 produced by* Paenibacillus polymyxa *[15, 16], bacteriocins OR-7 and L-1077 produced by* Lactobacillus salivarius* [17, 18], and bacteriocins E-760 and E 50-52 produced by* Enterococcus* spp. [19, 20]. All these bacteriocins have been reported to be active against Gram-positive and Gram-negative bacteria including* Campylobacter* spp., reducing* Campylobacter* colonization in poultry and considered potentially useful towards on-farm control of this foodborne human pathogen [21]. Most animal studies suggest that these bacteriocins considerably reduce* C. jejuni* colonization in chicken intestine and thus may reduce* Campylobacter* spp. infections in humans [17, 19, 20]. However, to our knowledge, none of the genes encoding these bacteriocins have been sequenced so far. Accordingly, in this study we report the use of synthetic genes designed from the published amino acid sequence of the mature bacteriocins SRCAM 602, OR-7, E-760, and L-1077 and with adapted codon usage for expression by* P. pastoris*, their cloning into the protein expression vector pPICZ*α*A, and their expression by recombinant* P. pastoris* X-33.

## 2. Materials and Methods

### 2.1. Microbial Strains and Plasmids

Microbial strains and plasmids used in this study are listed in Table 1.* Enterococcus faecium* T136 and* Pediococcus damnosus* CECT4797 were grown in MRS broth (Oxoid Ltd., Basingstoke, UK) at 32°C.* P. pastoris* X-33 (Invitrogen S.A., Barcelona, Spain) was cultured in YPD medium (Sigma-Aldrich Inc., St. Louis, MO, USA) at 30°C with shaking (200–250 rpm).* Escherichia coli* JM109 (Promega, WI, USA) was grown in LB broth (Sigma-Aldrich) at 37°C with shaking (250 rpm).* Listeria monocytogenes* CECT4032 was grown in LB at 37°C and* Salmonella typhimurium* CECT443 was grown in TSB (Oxoid) at 37°C.* Campylobacter jejuni* ATCC33560 and* C. jejuni* NCTC11168 were grown in BHI supplemented with 1% defibrinated horse serum (BD Bioscience, CA, USA) at 37°C in microaerophilic conditions.* E. coli* O157:H7 was grown in LB at 37°C with shaking (250 rpm).* Yersinia ruckeri* LMG3279 was grown in TSB at 28°C. Zeocin (Invitrogen) was added when needed at concentrations of 25, 100, or 1000 *μ*g/mL. Strains cited as CECT belong to the Colección Española de Cultivos Tipo (Valencia, Spain), ATCC to the American Type Culture Collection (Rockville, MD, USA), and NCTC to the National Collection of Type Cultures (London, UK).

### 2.2. Basic Genetic Techniques and Enzymes

The published amino acid sequences of mature bacteriocins SRCAM 602, OR-7, E-760, and L-1077 were used as templates to design the nucleotide sequence of the synthetic genes* srcam602, or-7, e-760, *and* l-1077 *matching the codon usage of* P. pastoris* X-33. These synthetic genes contained a 5′-nucleotide appendix including a* Xho*I restriction site and a 3′-nucleotide appendix including the termination of translation codon (TAA) and the* Not*I restriction site. All synthetic genes were supplied by GeneArt (Life Technologies, Paisley, UK). DNA restriction enzymes were supplied by New England BioLabs (Ipswich, MA, USA). Ligations were performed with the T4 DNA ligase (Roche Molecular Biochemicals, Mannheim, Germany).* E. coli*  JM109 cells were transformed as described by the supplier. Competent* P. pastoris* X-33 cells were obtained as recommended by the supplier and electroporation of competent cells was performed as previously described [22]. Electrocompetent cells were transformed with a Gene Pulser and Pulse Controller apparatus (Bio-Rad Laboratories, Hercules, CA, USA).

### 2.3. PCR Amplification and Nucleotide Sequencing

Oligonucleotide primers were obtained from Sigma-Genosys Ltd. (Cambridge, UK). PCR amplifications were performed in 50 *μ*L reaction mixtures containing 1 *μ*L of purified DNA, 70 pmol of each primer, and 1 U of Platinum Pfx DNA Polymerase (Invitrogen) in a DNA thermal cycler Techgene (Techne, Cambridge, UK). The PCR-generated fragments were purified by a NucleoSpin Extract II Kit (Macherey-Nagel GmbH & Co., Düren, Germany) for cloning and nucleotide sequencing. Nucleotide sequencing of the purified PCR products was performed using the ABI PRISM BigDye Terminator cycle sequence reaction kit and the automatic DNA sequencer ABI PRISM, model 377 (Applied Biosystems, Foster City, CA, USA), at the Unidad de Genómica, Facultad de Ciencias Biológicas, Universidad Complutense de Madrid (UCM), Madrid, Spain.

### 2.4. Cloning of the* srcam602, or-7, e-760, *and* l-1077* Synthetic Genes in* P. pastoris* X-33 and Antimicrobial Activity of the Transformants

The primers and inserts used for construction of the recombinant plasmids are listed in Table 2. Derivatives of plasmid pPICZ*α*A were constructed as follows: primers S602-F, S071-F, and SARP-R were used for PCR amplification from plasmids pMATSRCAM602, pMATOR-7, pMATE-760, and pMATL-1077 of nucleotide fragments in frame with the* S. cerevisiaeα*-factor secretion signal, without the Glu-Ala spacer adjacent to the Kex2 protease cleavage site, fused to the* srcam602*,* or-7*,* e-760,* and* l-1077* synthetic genes. Digestion of the above cited fragments with the* Xho*I-*Not*I restriction enzymes permitted ligation of the resulting R-SRCAM602, R-OR7, R-E760, and R-L1077 nucleotide fragments of 136-, 181-, 242-, and 167-bp, respectively, into pPICZ*α*A digested with the same enzymes to generate the plasmid-derived vectors pSRCAM602, pOR-7, pE-760, and pL-1077, respectively. Competent* E. coli* JM109 cells were used for cloning and vector propagation and the resulting transformants were confirmed by PCR amplification and sequencing of the inserts. Subsequently, the* Sac*I-linearized pSRCAM602, pOR-7, pE-760, and pL-1077 vectors were transformed into competent* P. pastoris* X-33 cells yielding zeocin resistant derivatives on YPD agar supplemented with zeocin (100 and 1,000 *μ*g/mL) and sorbitol (1 M). The presence of the integrated synthetic genes in the transformed yeast cells was confirmed by PCR and DNA sequencing of the inserts.

The antimicrobial activity of individual* P. pastoris* X-33SRCAM602,* P. pastoris* X-33OR-7,* P. pastoris* X-33E-760, and* P. pastoris* X-33L-1077 was screened by a streak-on-agar test (SOAT). Briefly, the* P. pastoris* transformants were streaked onto BMMY buffered methanol complex medium (1% yeast, 2% peptone, 100 mM potassium phosphate (pH 6), 1,34% yeast nitrogen base (YNB) without amino acids, 4 × 10^−5^% biotin, 0.5% methanol) agar and grown at 30°C to induce production of the bacteriocins. After incubation of the plates at 30°C during 24 h, 40 mL of MRS soft-agar containing 10^5^ cfu/mL of the indicator microorganism* Pediococcus damnosus* CECT4797 was added to the plates that were further incubated at 32°C for 24 h inhibition halos visualization.

### 2.5. Purification of the Antimicrobial Activity of Supernatants from the Recombinant Yeasts

The antimicrobial activity of supernatants from* P. pastoris* X-33 and* P. pastoris* X-33 (pPICZ*α*A) and the recombinant* P. pastoris* X-33SRCAM602,* P. pastoris* X-33OR-7,* P. pastoris* X-33E-760, and* P. pastoris* X-33L-1077 was purified using a previously described procedure [12]. Briefly, supernatants from early stationary phase 0.5 L cultures of the recombinant yeasts, grown in BMMY buffered methanol complex medium at 30°C, were precipitated with ammonium sulfate, desalted by gel filtration, and subjected to cation exchange-chromatography, followed by hydrophobic interaction-chromatography and reverse phase-chromatography in a fast protein liquid chromatography system (RP-FPLC) (GE Healthcare, Barcelona, Spain). The antimicrobial activity of the purified fractions was evaluated against* Pediococcus damnosus* CECT4797 by the microtiter plate assay (MPA).

### 2.6. Mass Spectrometry Analysis of Purified Supernatants

For determination of the molecular mass of peptides in supernatants of the recombinant yeasts, the eluted purified fractions with antimicrobial activity were subjected to matrix-assisted laser desorption/ionization time-of-flight mass spectrometry (MALDI-TOF MS). Briefly, 1 *μ*L samples were spotted onto a MALDI target plate and allowed to air-dry at room temperature. Then, 0.4 *μ*L of a 3 mg/mL of *α*-cyano-4-hydroxy-transcinnamic acid matrix (Sigma) in 50% acetonitrile were added to the dried peptide to digest spots and allowed again to air-dry at room temperature. MALDI-TOF MS analyses were performed in a 4800 Proteomics Analyzer MALDI-TOF/TOF mass spectrometer (Applied Biosystems, Framingham, MA), operated in 1 KV reflector mode. All mass spectra were calibrated externally using a standard peptide mixture (AB Sciex, MA, USA).

To determine the amino acid sequence of the purified peptides, the eluted purified fractions with antimicrobial activity were further subjected to MALDI-TOF/TOF tandem mass spectrometry. The samples were reduced, alkylated, digested with trypsin [23], and analysed in a 4800 Proteomics Analyzer MALDI-TOF/TOF mass spectrometer (Applied Biosystems). The acquisition method for MS analysis was 1 KV reflector positive mode. Peptides from the MS spectra were manually selected for fragmentation analysis. The acquisition method for MS/MS analysis was MS/MS-1 KV in reflector positive mode with CID for fragmentation. The collision gas was atmospheric and the precursor mass window was ±5 Da. The plate model and default calibration were optimized for the MS/MS spectra processing. The parameters used to analyze the data were signal to noise = 20, resolution >6000. For protein identification, the* de novo* amino acid sequence from the fragmentation spectra of selected peptides was performed using the De Novo tool software (Applied Biosystems), and tentative sequences were manually checked and validated. Homology searches of the deduced amino acid sequences were performed through the NCBI/Blast. All mass spectrometry (MS) analyses were performed at the Unidad de Proteómica, Facultad de Farmacia, Universidad Complutense de Madrid (UCM), Madrid, Spain.

### 2.7. Antimicrobial Activity of Purified Supernatants from the Recombinant Yeasts

The antimicrobial activity of purified supernatants from the recombinant* P. pastoris* X-33SRCAM602,* P. pastoris* X-33OR-7,* P. pastoris* X-33E-760, and* P. pastoris* X-33L-1022 was evaluated against* Listeria monocytogenes* CECT4032,* E. coli* O157:H7,* Yersinia ruckeri* LMG3279,* Campylobacter jejuni* ATCC33560, and* C. jejuni* NCTC11168 by using a MPA, as previously described [12].

## 3. Results 

### 3.1. Cloning of Synthetic Genes Encoding Bacteriocins and Their Expression by Recombinant* P. pastoris*

The cloning of PCR-amplified fragments from plasmids encoding synthetic genes designed from the published amino acid sequence of the mature bacteriocins SRCAM 602, OR-7, E-760, and L-1077 into the protein expression vector pPICZ*α*A resulted in the recombinant plasmids pSRCAM602, pOR-7, pE-760, and pL-1077 (Table 1). Similarly, transformation of the linearized plasmids into competent* P. pastoris* X-33 permitted isolation of the* P. pastoris* X-33SRCAM602 (*srcam602*),* P. pastoris* X-33OR-7 (*or-7*),* P. pastoris* X-33E-760 (*e-760*), and* P. pastoris* X-33L-1077 (*l-1077*) recombinants. However, none of the recombinant yeasts showed direct antimicrobial activity against* P. damnosus* CECT4797, even those recombinant yeasts selected for their high zeocin resistance (1,000 *μ*g/mL). Colonies of* P. pastoris* X-33 and* P. pastoris* X-33 (pPICZ*α*A) were used as bacteriocin-negative controls to discard the possibility that the antimicrobial activity possibly observed was due to metabolites other than bacteriocins.

### 3.2. Purification of the Antimicrobial Activity of Supernatants from the Recombinant* P. pastoris* and Mass Spectrometry Analysis

**Figure 1:**
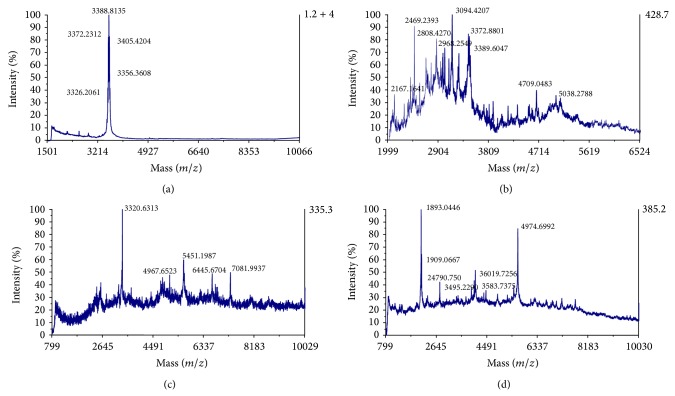
Mass spectrometry analysis of purified supernatants from* P. pastoris* X-33SRCAM602 (a),* P. pastoris* X-33OR-7 (c),* P. pastoris* X-33E-760 (b), and* P. pastoris* X-33L-1077 (d), eluted after RP-FPLC. Numbers indicate the molecular mass in daltons of most of the observed peptide fragments.

When supernatants from* P. pastoris* X-33SRCAM602,* P. pastoris* X-33OR-7,* P. pastoris* X-33E-76, and* P. pastoris* X-33L-1077 were purified by a multistep chromatographic procedure, only eluted fractions after the hydrophobic interaction-chromatography step showed full antimicrobial activity against* Pediococcus damnosus* CECT4797 as the indicator microorganism (Table 4). MALDI-TOF MS analysis of eluted fractions after the RP-FPLC step showed that supernatants from* P. pastoris* X-33SRCAM602 showed a major peptide fragment of 3388.8 Da (Figure 1(a)). However, the purified supernatant from* P. pastoris* X-33OR-7 showed a major peptide fragment of 3094.4 Da and peptide fragments of major and minor molecular mass (Figure 1(b)). Similarly, a large display of peptide fragments of different molecular masses was observed in the purified supernatants from* P. pastoris* X-33E-760 (Figure 1(c)) and* P. pastoris* X-33L-1077 (Figure 1(d)). MALDI-TOF MS/MS spectrometry analysis of trypsin-digested peptides from purified supernatants from all recombinant* P. pastoris,* determined that none of the most probable or* de novo* amino acid sequence of the evaluated peptide fragments matched the expected amino acid sequence of the bacteriocins SRCAM 602, OR-7, E-760, and L-1077 (Table 5). However, some of the determined peptide fragments were homologous to those observed in proteins from ABC-transporter systems, histidine kinase protein family, peptidase C1 superfamily, and nucleosome binding proteins.

### 3.3. Antimicrobial Activity of Purified Supernatants from the Recombinant Yeasts

The purified supernatants from recombinant* P. pastoris*, constructed for expression of the bacteriocins SRCAM 602, OR-7, E-760, and L-1077 showed antimicrobial activity against* Pediococcus damnosus* CECT4797 but did not display a measurable antimicrobial activity against* L. monocytogenes* CECT4032,* E. coli* O157:H7,* Y. ruckeri* LMG3279,* C. jejuni* ATCC33560, and* C. jejuni* NCTC11168, when evaluated by a microtiter plate assay (MPA). The purified supernatants from* P. pastoris* X-33 and* P. pastoris* X-33 (pPICZ*α*A) did not show antagonistic activity neither against* Pediococcus damnosus* CECT4797 nor against any of the indicator bacteria cited above.

## 4. Discussion

To circumvent the proliferation of emerging pathogenic and antibiotic-resistant bacteria, bacteriocins produced by LAB emerge as natural antimicrobial peptides with potential applications in food preservation, livestock protection, and medical applications [1, 24]. However, the high cost of synthetic bacteriocin synthesis, their low yields, and the production of potential virulence factors from some natural bacterial producers drive the exploration of microbial systems for the biotechnological production of bacteriocins by heterologous LAB and yeasts [6, 8]. Furthermore, the use of synthetic genes may become a useful tool for production and functional expression of bacteriocins by heterologous microbial hosts [12].

The development of heterologous expression systems for bacteriocins may offer a number of advantages over native systems, facilitating the control of bacteriocin gene expression or achieving higher production levels. Although a number of yeast platforms have been used for the production of peptides and proteins, including bacteriocins [10, 11, 25], the use of synthetic genes has been only barely explored for their expression by recombinant yeasts [12]. In this study, the protein expression vector pPICZ*α*A containing an strong and inducible promoter and the Kex2 signal cleavage site for processing of fusion proteins [26] has been used to drive the expression of synthetic genes encoding the mature bacteriocins SRCAM 602, OR-7, E-760, and L-1077, by recombinant* P. pastoris* X-33 derivatives.

Initial results with* P. pastoris* X-33SRCAM602,* P. pastoris* X-33OR-7,* P. pastoris* X-33E-760, and* P. pastoris* X-33L-1077 determined that none of the recombinant yeasts, encoding synthetic genes for expression of the cloned bacteriocins, showed antimicrobial activity when individual colonies were screened by a streak-on-agar test (SOAT). Highly variable yields of secreted proteins have been achieved using the* P. pastoris* expression system and cases of low secretory yields or complete failure in protein production have also been reported [3, 27, 28]. A number of factors may affect the production of foreign peptides by heterologous yeasts including copy number integration of the expression vectors in the yeast DNA, mRNA stability, errors in mRNA translation, uncoordinated rates of protein synthesis, folding and translocation, and undesired proteolysis of heterologous proteins by resident proteases or by proteases in the extracellular space being secreted, cell-wall associated, or released into the culture medium as a result of cell disruption [29–31]. The use of the* P. pastoris* expression system for overproduction of peptides and proteins is known to be somewhat hampered by its unpredictable yields of production of heterologous proteins, which is now believed to be caused in part by their varied efficiencies to traffic through the host secretion machinery [3, 28].

The amino acid sequence following the Kex2 secretion signal may also interfere with the secretion of fused peptides or proteins by recombinant* P. pastoris*. Furthermore, the yields of many recombinant proteins seem to be influenced by the Kex2 P1′ site residue [3]. In this study, the Kex2 P1′ site residues for mature SRCAM 602, OR-7, E-760, and L-1077 were the amino acids A, K, N, and T, respectively (Table 3). However, the cloning in* P. pastoris* of the bacteriocins enterocin A (EntA) and enterocin P (EntP) with the Kex2 P1′ site residues A and T, respectively, showed an overproduction of both bacteriocins over their natural producers and an intense antimicrobial activity when colonies of the recombinant yeasts were evaluated by the SOAT [11, 22].

The use of a multistep chromatographic procedure for purification of the expected antimicrobial activity in supernatants of the recombinant* P. pastoris* determined that only eluted fractions after the hydrophobic interaction-chromatography step showed full antimicrobial activity (Table 4), probably due to removal of antimicrobial inhibitors, disaggregation of the bacteriocins, or changes in conformation of the bacteriocins in the hydrophobic solvent. The antimicrobial activity of the supernatant from* P. pastoris* X-33SRCAM602 was much higher than that of the rest of the recombinant* P. pastoris*. While being interesting, this was not an unexpected observation since purification of the circular bacteriocin garvicin ML, produced by* Lactococcus garvieae* DCC43, showed a higher antibacterial activity and a broader antimicrobial spectrum as it was increasingly purified [32]. However, MALDI-TOF MS analysis of the purified supernatants from* P. pastoris* X-33SRCAM602,* P. pastoris* X-33OR-7,* P. pastoris* X-33E-760, and* P. pastoris* X-33L-1077 mostly showed a large display of peptide fragments of different molecular mass than deduced from the calculated molecular mass of the cloned bacteriocins (Figure 1). The different molecular mass of the resulting peptide fragments may suggest the existence of truncated bacteriocins, the interaction of the bacteriocins with unknown biological compounds or the bacteriocins being subjected to posttranslational modifications (PTM) such as phosphorylation, acetylation, methylation, oxidation, formylation, disulfide bond formation, and N-linked and O-linked glycosylation [8, 33]. The presence of cysteine residues in the bacteriocins SRCAM 602, OR-7, and E-760 would permit the formation of disulfide bridges but also permits its oxidation, glutathionylation, and cysteinylation. The absence in all cloned bacteriocins of attachment sites for N-linkages precludes its N-glycosylation, but the presence of threonines and serines makes the bacteriocins sensitive to O-glycosylation [8, 12, 33].

However, MALDI-TOF MS/MS analysis of trypsin-digested peptides from purified supernatants from all recombinant* P. pastoris* determined that none of the evaluated peptide fragments matched the expected amino acid sequence of the bacteriocins SRCAM 602, OR-7, E-760, and L-1077 (Table 5). From the results obtained it may be suggested that very low yields of secreted and/or purified bacteriocins are obtained after cloning of synthetic genes encoding the bacteriocins SRCAM 602, OR-7, E-760, and L-1077 in* P. pastoris*. This observation was also not unexpected because bacteriocins cloned into* Saccharomyces cerevisiae*,* P. pastoris*,* Kluyveromyces lactis*,* Hansenula polymorpha,* and* Arxula adeninivorans* have been produced with variable success regarding their production, secretion, and functional expression [11]. Furthermore, since one of the main bottlenecks in recombinant protein production is the inability of foreign peptides to reach their native conformation in heterologous yeast hosts, it could also happen that incorrectly folded SRCAM 602, OR-7, E-760, and L-1077 are accumulated in the endoplasmic reticulum (ER) of recombinant* P. pastoris*, activating the unfolded protein response (UPR) and the ER-associated degradation (ERDA) of the misfolded bacteriocins, leading to persistent ER stress conditions causing much lower efficiencies to traffic through the host secretion machinery, apoptosis, and cell death [8, 28, 34, 35]. In any case, the inconsistent secretory productivity among recombinant proteins has always been a major obstacle for routine application of* P. pastoris* as an eukaryotic protein expression system in both research and industry [3].

The purified supernatants from the recombinant* P. pastoris* constructed for expression of the bacteriocins SRCAM 602, OR-7, E-760, and L-1077 showed a measurable antimicrobial activity against* Pediococcus damnosus* CECT4797 (Table 4), but not against* L. monocytogenes* CECT4032,* E. coli* O157:H7,* Y. ruckeri* LMG3279,* C. jejuni* ATCC33560, and* C. jejuni* NCTC11168. One of the remarkable features of bacteriocins is that they are very potent, being active in nanomolar concentrations, thereby surpassing by about 1,000-fold the activity of AMPs produced by eukaryotic cells [36]. One of the major reasons for this extreme potency is that bacteriocins apparently recognize specific receptors on target cells, while the interactions between AMPs and microorganisms are mostly nonspecific. Furthermore, the target receptor for class IIa bacteriocins has been identified as proteins of the sugar transporter mannose phosphotransferase system (Man-PTS), with the most potent receptors being those found in* Listeria* spp. [37, 38]. However, the very low yields of secreted or purified bacteriocins in supernatants of the recombinant* P. pastoris* may be responsible for their nondetected antilisterial activity.

It could be also hypothesized that peptide fragments aggregated to or coeluting with the purified bacteriocins may be responsible for the antimicrobial activity of the eluates against the sensitive indicator* Pediococcus damnosus* CECT4797, but not against any other of the indicator bacteria tested. Many proteins contain encrypted within their primary structure bioactive peptides with antimicrobial activity following hydrolytic release from the native molecule [39, 40]. Incorrect disulfide bond formation, misfolding of the secreted bacteriocin and extensive PTMs were suggested to be responsible for the lower antilisterial activity and the nonmeasurable antimicrobial activity against Gram-negative bacteria of the bacteriocin E 50-52 (BacE50-52), produced by recombinant* P. pastoris* X-33BE50-52S and* K. lactis* GG799BE50-52S [12]. This bacteriocin, originally produced by* Enterococcus faecium* B-30746, was also reported to display a high and broad antimicrobial activity against Gram-positive and Gram-negative bacteria, including* Campylobacter* spp. [20, 41].

The correct processing, secretion and functional expression of the bacteriocins EntP [22], hiracin JM79 (HirJM79) [42] and EntA [11], produced by recombinant yeasts, contrast with the low biological activity of the sakacin A (SakA) and the chimera EntP/SakA, produced by recombinant* P. pastoris* and* K. lactis* producers [8]. Misfolding of SakA and EntP/SakA and induction of the yeasts' UPR may be responsible for apoptosis in recombinant* P. pastoris* producers of SakA and for extensive PTMs in recombinant* P. pastoris* and* K. lactis*, producers of SakA and EntP/SakA [8]. These results, obtained by our research group, also contrast with the low antimicrobial activity against* Pediococcus damnosus* CECT4797 and the absence of antimicrobial activity against Gram-positive and Gram-negative bacteria of the purified supernatants from recombinant* P. pastoris* encoding the mature bacteriocins SRCAM 602, OR-7, E-760, and L-1077.

Nevertheless, it should be also worth to notice that bacteriocins SRCAM 602 [16], OR-7 [17], E-760 [19], and L-1077 [18], although being reported as bacteriocins with a broad antimicrobial activity against Gram-positive and Gram-negative microorganisms including* Campylobacter* spp., have not been fully characterized at their biochemical and genetic level, the genetic identification of their structural and adjacent genes has not been yet reported, and their molecular masses, deduced from their reported amino acid sequences, were not identical to the experimentally determined molecular mass from the purified bacteriocins [16–19]. Furthermore, recent reports suggest that bacteriocin SRCAM 602, previously reported to be produced by* Paenibacillus polymyxa* NRRL B-30509 and claimed to be responsible for inhibition of* C. jejuni*, could not be detected in the purified supernatants of the producer strain while the* srcam602* structural gene was not found via a PCR-based approach using degenerate nucleotide primers or by genomic sequencing of the bacteriocin producer [43]. Similarly,* Bacillus circulans* (now* Paenibacillus terrae*) NRRL B-30644 previously reported to produce SRCAM 1580, a bacteriocin active against* C. jejuni* [16], has been recently suggested not to produce this bacteriocin and no genetic determinants for its production were shown. Instead the anti-*Campylobacter* activity of this strain is due to the production of the lipopeptide tridecaptin A1 whereas this strain also produces the novel lantibiotic paenicidin B, active against Gram-positive bacteria [44].

Although the cloning in recombinant yeasts of synthetic genes encoding bacteriocins drives the production, antimicrobial activity, and specific antimicrobial activity of the cloned bacteriocins in the absence of dedicated immunity and secretion proteins [12], these results contrast with the negligible antimicrobial activity against Gram-positive and Gram-negative bacteria of purified supernatants from recombinant* P. pastoris* encoding the bacteriocins SRCAM 602, OR-7, E-760, and L-1077. Accordingly, since production of bacteriocins from synthetic bacteriocin genes is difficult to predict, further efforts should be performed for a more efficient genetically engineered production and functional expression of other bacteriocin synthetic genes or their chimeras by heterologous producer yeasts. The design of further and novel successful genetic approaches for production and functional expression of bacteriocins by yeasts would facilitate their biotechnological applications as natural antimicrobial agents in food, animal husbandry, and medicine.

## Figures and Tables

**Table 1:** Bacterial strains and plasmids used in this study.

Strain or plasmid	Description^a^	Source and/or reference^b^
Strains		
*Enterococcus faecium* T136	Enterocin A and B producer; MPA positive control	DNBTA
*Pediococcus damnosus* CECT4797	MPA indicator microorganism	CECT
*Escherichia coli* JM109	Selection of recombinant plasmids	Promega
*Pichia pastoris *X-33	Yeast producer	Invitrogen Life Technologies
Plasmids		
pMA-T	Amp^r^; carrier of synthetic genes	GeneArt Life Technologies
pPICZ*α*A	Zeo^r^; integrative plasmid carrying the secretion signal sequence from the *S. cerevisiaeα*-factor prepropeptide and functional sites for integration at the 5′ AOX1 locus of *P. pastoris* X-33	Invitrogen Life Technologies
pMATSRCAM602	Amp^r^; pMA-T plasmid carrying the *srcam602* synthetic gene with the *P. pastoris* codon usage	GeneArt Life Technologies
pMATOR-7	Amp^r^; pMA-T plasmid carrying the *or-7* synthetic gene with the *P. pastoris *codon usage	GeneArt Life Technologies
pMATE-760	Amp^r^; pMA-T plasmid carrying the *e-760* synthetic gene with the *P. pastoris *codon usage	GeneArt Life Technologies
pMATL-1077	Amp^r^; pMA-T plasmid carrying the *l-1077* synthetic gene with the *P. pastoris *codon usage	GeneArt Life Technologies
pSRCAM602	pPICZ*α*A derivative with the *srcam602* synthetic gene	This work
pOR-7	pPICZ*α*A derivative with the *or-7* synthetic gene	This work
pE-760	pPICZ*α*A derivative with the *e-760* synthetic gene	This work
pL-1077	pPICZ*α*A derivative with the *l-1077* synthetic gene	This work

^a^Amp^r^: ampicillin resistance; Zeo^r^: zeocin resistance.

^b^DNBTA: Departamento de Nutrición, Bromatología y Tecnología de los Alimentos, Facultad de Veterinaria, Universidad Complutense de Madrid (Madrid, Spain); CECT: Colección Española de Cultivos Tipo Valencia, Spain).

**Table 2:** Primers and PCR products used in this study.

Primers, PCR products, or bacteriocins	Nucleotide sequence (5′-3′) or description	Amplifications
Primers		
S602-F	GCCATGAGCTCGAATTCTCGAGAAAAG	R-SRCAM602, R-E760, R-L1077
S071-F	GTCCAGAGCTCGAATTCTCGAGAAAAG	R-SRCAM 602, R-E760, R-L1077, R-OR7
SARP-R	AGGTACCATAAGTTGCGGCCGC	R-OR7
ALFA-F	TACTATTGCCAGCATTGCTGC	pPICZ*α*A amplification fragment including the cloned synthetic gene
3AOX1-R	GCAAATGGCATTCTGACATCC	pPICZ*α*A amplification fragment including the cloned synthetic gene
PCR products		
R-SRCAM602	136-bp *Xho*I/*Not*I fragment containing the *α*-factor Kex2 signal cleavage fused to the mature synthetic *srcam602* gene with the *P. pastoris* codon usage	
R-OR7	181-bp *Xho*I/*Not*I fragment containing the *α*-factor Kex2 signal cleavage fused to the mature synthetic *or-7* gene with the *P. pastoris* codon usage	
R-E760	242-bp *Xho*I/*Not*I fragment containing the *α*-factor Kex2 signal cleavage fused to the mature synthetic *e-760* gene with the *P. pastoris* codon usage	
R-L1077	167-bp *Xho*I/*Not*I fragment containing the *α*-factor Kex2 signal cleavage fused to the mature synthetic *l-1077* gene with the *P. pastoris* codon usage	
Bacteriocins		
BacSRCAM602 (amino acid sequence)	ATYYGNGLYCNKQKHYTWVDWNKASR EIGKITVNGWVQH	
BacSRCAM602 (*P. pastoris* codon usage)	gctacttactacggtaacggtctttactgtaacaagcagaagcact acacttgggttgactggaacaaggcttccagagagatcggtaag atcactgttaacggttgggttcaaca	
BacOR-7 (amino acid sequence)	KTYYGTNGVHCTKNSLWGKVRLKNMK YDQNTTYMGRLQDILLGWATGAFGKTFH
BacOR-7 (*P. pastoris* codon usage)	aagacttactacggaactaacggtgttcactgtactaagaattcctt gtggggtaaggttagattgaagaacatgaagtacgaccagaaca ctacttacatgggtagattgcaggacatcttgttgggttgggctact ggtgctttcggtaagacttttcat
BacE-760 (amino acid sequence)	NRWYCNSAAGGVGGAAVCGLAGYVGE AKENIAGEVRKGWGMAGGFTHNKACKS FPGSGWASG
BacE-760 (*P. pastoris* codon usage)	aacagatggtactgtaactccgctgctggtggtgttggtggtgct gctgtttgtggtttggctggttatgttggtgaggctaaagaaaacat tgctggtgaggttagaaagggttggggtatggctggtggtttcac tcataacaaggcttgtaagtccttcccaggttctggttgggcttctggt
BacL-1077 (amino acid sequence)	TNYGNGVGVPDAIMAGIIKLIFIFNIRQGY NFGKKAT
BacL-1077 (*P. pastoris *codon usage)	actaactacggtaacggtgttggtgttccagacgctattatggctg gtatcatcaagttgatcttcatcttcaacatcagacagggttacaac ttcggtaagaaggctact

**Table 3:** Amino acid sequence of cloned bacteriocins and other class IIa bacteriocins.

Bacteriocin	Amino acid sequence	Number of amino acids
SRCAM 602	ATYYGNGLYCNKQKHYTWVDWNKASREIGKITVNGWVQH	39
OR-7	KTYYGTNGVHCTKNSLWGKVRLKNMKYDQNTTYMGRLQDILLGW ATGAFGKTFH	54
E-760	NRWYCNSAAGGVGGAAVCGLAGYVGEAKENIAGEVRKGWGMAGG FTHNKACKSFPGSGWASG	62
E 50-52	TTKNYGNGVCNSVNWCQCGNVWASCNLATGCAAWLCKLA	39
L-1077	TNYGNGVGVPDAIMAGIIKLIFIFNIRQGYNFGKKAT	37
EntP	ATRSYGNGVYCNNSKCWVNWGEAKENIAGIVISGWASGLAGMGH	44
EntA	TTHSGKYYGNGVYCYKNKCTVDWAKATTCIAGMSIGGFLGGAI PGKC	47
HirJM79	ATYYGNGLYCNKEKCWVDWNQAKGEIGKIIVNGWVNHGPWAP RR	44
SakA	ARSYGNGVYCNNKKCWVNRGEATQSIIGGMISGWASGLAGM	41

Underlined, the class IIa N-terminal consensus sequence YGNGV(X)C.

**Table 4:** Antimicrobial activity of fractions generated during purification of supernatants from *P. pastoris* X-SRCAM602, *P. pastoris* X-33OR-7, *P. pastoris* X-33E-760, and *P. pastoris* X-33L-1077, grown in BMMY with methanol.

Strain	Antimicrobial activity (BU/mL) of the purified fractions^a^					
	SN	AS	GF	SE	OE	RP-FPLC
*P. pastoris* X-33SRCAM602	NA	NA	NA	NA	12,800	1,106,531
*P. pastoris* X-33OR-7	NA	NA	127	32	1,391	10,027
*P. pastoris* X-33E-760	NA	NA	NA	NA	687	14,442
*P. pastoris* X-33L-1077	NA	NA	35	23	2,069	13,213

Most of the data are mean from two independent determinations in triplicate.

^a^Antimicrobial activity against *Pediococcus damnosus* CECT4797 as determined by microtiter plate assay (MPA). BU: bacteriocin units. NA: no activity.

Purification fractions: SN: supernatant; AS: ammonium sulfate precipitation; GF: gel filtration; SE: Sepharose fast flow eluate; OE: Octyl Sepharose eluate; RP-FPLC: reversed-phase eluate.

**Table 5:** Results obtained by MALDI-TOF/TOF MS analysis of the eluted RP-FPLC fractions from purified supernatants of the recombinant *P. pastoris *X-33 derivatives.

Bacteriocin	Predicted molecular mass (Da)	Trypsin-digested precursor (Da)	Amino acid sequence (MS/MS analysis)
SRCAM 602	4,630.1	926.65 1,500.95 1,614.97	SANALRPPT GDKENAAKASSVPAR KTGGNRAVSGAGEIAAR
OR-7	6,215.1	Deficient signal	—
E-760	6,179.8	1,166.63 1,579.68	VGNPLHGIFGR SLSAYMFFANEQR
L-1077	4,002.6	1,638.76 1,718.73	IVGSQAGIGEYLFER LVELSEQELVDCER
